# Commentary: Cat exposure and asthma outcomes in a cohort of children with asthma and allergy

**DOI:** 10.3389/falgy.2026.1997917

**Published:** 2026-10-16

**Authors:** Milad Ameri, Pål Johansen, Marie-Charlotte Brüggen

**Affiliations:** 1 Faculty of Medicine, University of Zurich, Zurich, Switzerland; 2 Department of Dermatology, University Hospital Zurich (USZ), Zurich, Switzerland; 3 Christine Kühne Center for Allergy Research and Education, Davos, Switzerland

**Keywords:** allergen avoidance, asthma, cat allergy, confounding by indication, exposure misclassification, human–animal bond, pet attachment, register-based epidemiology

## Introduction

Putri et al. (1) reported that among 30,277 Swedish children with established asthma and airway allergy, parental cat ownership was not associated with asthma exacerbation, medication-defined severity, asthma control, or lung function. The study is unusually comprehensive: validated diagnostic algorithms, a national register of cats, five outcome dimensions, and a probabilistic bias analysis for exposure misclassification. Its central message — that blanket advice to avoid cats may not be uniformly beneficial — is well supported and clinically important.

We wish to develop an issue the authors raise and then, understandably, leave. Our central point is that their exposure variable, cat ownership, is a decision, not a dose: it reflects which families keep a cat rather than how much allergen a child receives. Once this is taken seriously, the behavior of families ceases to be background noise and becomes part of what the estimate is made of. We describe three consequences — selection into ownership, misclassification of exposure, and the analytic role of behavioral constructs — indicate the likely direction of bias, distinguish hypotheses from findings, and argue that the remedy is measurement rather than caution.

### Ownership is a decision, not a dose

Putri et al. note that residual confounding by indication cannot be excluded, since parents may avoid keeping a cat because of a child's allergy. The authors' data are consistent with this, with severe allergic rhinitis at baseline being less common among cat-exposed children (11.5% vs. 14.2%), despite comparable initial asthma severity and parental atopy.

The direction of this selection is not random. When physicians advise removal of a pet, most families decline. In a 1997 survey of 341 adults with diagnosed cat or dog allergy who had been advised specifically to stop sharing living quarters with their animal, 21.4% removed it or excluded it from the home; among a subset of 122 whose animal had since died, 70.5% had acquired another (2). Though dated and single-sample, this estimate is consistent with recent reviews describing avoidance as frequently insufficient (3). Families who relinquish a pet appear to be a minority and may represent a subgroup in whom allergic symptoms or medical advice have become sufficiently consequential to alter ownership. Cat-owning households may therefore represent a selected population, in which the presence of a cat has already proved acceptable, or at least tolerable, to the family. We hypothesize that this biases the association toward the null, by an amount register data cannot estimate.

### No cat at home is not the same as no cat exposure

The relevant comparator is not “no cat exposure” but “no cat in the household,” a substantially different construct. Children classified as living without a cat may nevertheless encounter substantial cat allergen through schools, childcare, relatives, and friends (3, 4). This was demonstrated in the same country by the senior author of the present study: among more than 400 asthmatic, cat-sensitized children with no cat at home, those attending classes where more than 18% of classmates owned cats showed decreased peak expiratory flow, more symptom days, and greater medication use once term began (5). If unrelated to outcome, this misclassification would attenuate the estimate toward the null.

A related issue concerns how ownership was ascertained. The National Cat Register was established in 2023, and the authors appropriately model incomplete ascertainment in their probabilistic bias analysis. Nevertheless, the cat-exposed group was markedly enriched for Nordic-born parents (mothers 92.2% vs. 78.9%; fathers 89.9% vs. 77.7%), illustrating that cat ownership is socially patterned. This does not demonstrate differential misclassification, but it raises the possibility that completeness of registration or linkage varies with sociodemographic or behavioral characteristics. Were these also related to asthma outcomes, misclassification could be differential, which can bias estimates in either direction (6) — a possibility that a bias analysis assuming non-differential ascertainment does not address. We raise this not to dispute the null finding, which was consistent across all five outcomes, but because the assumption is substantive and merits explicit interrogation.

### These constructs are measurable

The authors list “cat-keeping practice” and “cultural attitudes toward cats” as limits on generalizability. These are measurable, but their analytic use depends on their causal position (Table 1).

**Table 1:** Behavioral constructs that could complement household cat ownership, classified by position in the causal structure, expected effect on the ownership–asthma estimate, and measurement.

Construct	Position in causal structure	Expected effect on the estimate	Measurement (status)
Attachment to the animal	Determinant of ownership; confounder only if it affects outcome independently of cat exposure	Selective non-ownership by families of more reactive children: bias toward the null	LAPS, 23 items (7); Brief-LAPS, 7 items, >2,000 owners in three countries (8); 11-item version, invariant across four countries (9) (validated)
Cat-keeping and avoidance practice (bedroom access, indoor/outdoor status, washing, allergen-reducing diet)	Intermediate between ownership and allergen dose	If unmeasured: exposure misclassification, attenuation; if adjusted for: overadjustment	Structured exposure inventory; environmental Fel d 1 (10) (partially validated)
Symptom attribution	May be influenced by the outcome itself	Direction unpredictable; conditioning may introduce bias	Structured clinical history (11) (not formally validated)

Attachment to the animal determines ownership. It is a confounder only if it also affects asthma independently of cat exposure; otherwise it characterizes selection. Validated instruments include the Lexington Attachment to Pets Scale (7) and two brief versions (8, 9).

Cat-keeping and avoidance practices lie between ownership and allergen dose, so adjusting for them would overadjust; recorded by structured inventory and environmental Fel d 1 (10), they can instead grade exposure by dose. Symptom attribution (11) may itself reflect the child's asthma and is not a simple covariate.

## Discussion

The clinical implication follows from the authors' own conclusion. If general avoidance advice is not uniformly warranted, what replaces it is a conversation, and that conversation depends on knowing how attached the family is, what they already do, and what they are willing to do. This is no longer merely counseling: interventions targeting the cat rather than requiring removal of the animal — allergen-reducing diets and immunization of cats against Fel d 1 — have made “keep the cat, change the care pathway” a testable clinical strategy (10, 12, 13). Patient preference thereby becomes part of the clinical decision.

This Research Topic notes that avoidance of animal allergens is in most cases not possible because of the emotional impact. Putri et al. provide unusually thorough evidence that, for cats at least, this concern may be less consequential for asthma outcomes than has been feared (1). We propose that the field stop treating the human–animal bond as an unmeasured limitation and begin recording it. A brief validated scale, administered alongside sensitization testing, would make selection into ownership measurable rather than assumed, and would allow the field to ask a question that ownership data alone cannot answer: not simply whether cat-owning families differ in outcome, but whether any difference reflects allergen dose or selection into ownership — a question for explicit mediation analysis rather than covariate adjustment.
